# Pancreatic Cancer and Therapy: Role and Regulation of Cancer Stem Cells

**DOI:** 10.3390/ijms22094765

**Published:** 2021-04-30

**Authors:** Susmita Barman, Iram Fatima, Amar B. Singh, Punita Dhawan

**Affiliations:** 1 Department of Biochemistry and Molecular Biology, Omaha, NE 68198, USA; susmita.barman@unmc.edu (S.B.); iram.fatima@unmc.edu (I.F.); amar.singh@unmc.edu (A.B.S.); 2 VA Nebraska-Western Iowa Health Care System, Omaha, NE 68105, USA; 3 Buffet Cancer Center, University of Nebraska Medical Center, Omaha, NE 68105, USA

**Keywords:** MASTL, pancreatic cancer, chemoresistance

## Abstract

Despite significant improvements in clinical management, pancreatic cancer (PC) remains one of the deadliest cancer types, as it is prone to late detection with extreme metastatic properties. The recent findings that pancreatic cancer stem cells (PaCSCs) contribute to the tumorigenesis, progression, and chemoresistance have offered significant insight into the cancer malignancy and development of precise therapies. However, the heterogeneity of cancer and signaling pathways that regulate PC have posed limitations in the effective targeting of the PaCSCs. In this regard, the role for K-RAS, TP53, Transforming Growth Factor-β, hedgehog, Wnt and Notch and other signaling pathways in PC progression is well documented. In this review, we discuss the role of PaCSCs, the underlying molecular and signaling pathways that help promote pancreatic cancer development and metastasis with a specific focus on the regulation of PaCSCs. We also discuss the therapeutic approaches that target different PaCSCs, intricate mechanisms, and therapeutic opportunities to eliminate heterogeneous PaCSCs populations in pancreatic cancer.

## 1. Introduction

Pancreatic cancer (PC) is one of the most lethal cancers, which is usually diagnosed in advanced stages and has a five-year patient survival rate of less than 9% [1,2]. Based on the chemoresistance and lack of immunotherapy, combined with the tendency of the disease to spread early, it is projected that the PC will become the 2nd most cause of cancer-related death in the US by 2030 [3]. Age, alcohol, and chronic pancreatitis are known risk factors for the PC. However, they are not specific to the disease [4]. Pancreatic cancer is usually associated with KRAS/p53 mutations or overexpression of oncogenic receptor tyrosine kinases, such as EGFR, FGFR1, or IGF1 [5,6,7]. Currently, the main treatment approach is surgery coupled with chemo- or radiation therapy though it does not present satisfactory results. Most patients subjected to resection of the tumor will die from metastasis within five years [8]. Pancreatic ductal adenocarcinoma (PDAC) is one of the deadliest cancers in humans due to its late detection and high rate of metastatic features. Gemcitabine (a pyrimidine analog) is the first line of therapy in advanced PDAC for many years [9]. Unfortunately, it is effective in only 23.8% of PDAC cases, possibly due to the dense tumor stroma and, therefore, low diffusion of the drug [10,11].

A subpopulation of undifferentiated cancer stem cells is thought to mediate not only tumorigenicity but also resistance and metastasis to therapy [12]. Cancer stem cells appear to can resist common treatments, such as chemotherapy and radiotherapy, so it is critical to understand the mechanisms involved in this resistance [13]. However, a significant heterogeneity appears to exist in the cancer stem cell subpopulations [14,15,16,17]. Therefore, it is essential to understand the biological characteristics of the CSC subpopulations to track them throughout the treatment course. Furthermore, a better insight of the CSCs biology could direct to new therapeutic approaches at abrogating CSCs in PC. The current review highlights the information related to the CSCs in PC based on a comprehensive literature search in the hope of identifying innovative approaches for treating pancreatic cancer through targeting pancreatic cancer stem cells (PaCSCs).

### 1.1. Characteristic Features of Cancer Stem Cells

Cancer stem cells (CSCs) are a subset of cancer cells that have the capability to self-renew, cellular plasticity (differentiate into defined progenies), initiate and maintain tumor growth in vivo. CSCs also possess unique cell surface markers, which underscore their heterogeneity, abilities to alter their phenotype significantly in response to a stimulus or consequence of chemotherapy, and thus help create resilience to the treatment and evade the anticancer immunity [12,13,14,15,16,17,18,19,20].

**Figure 1:**
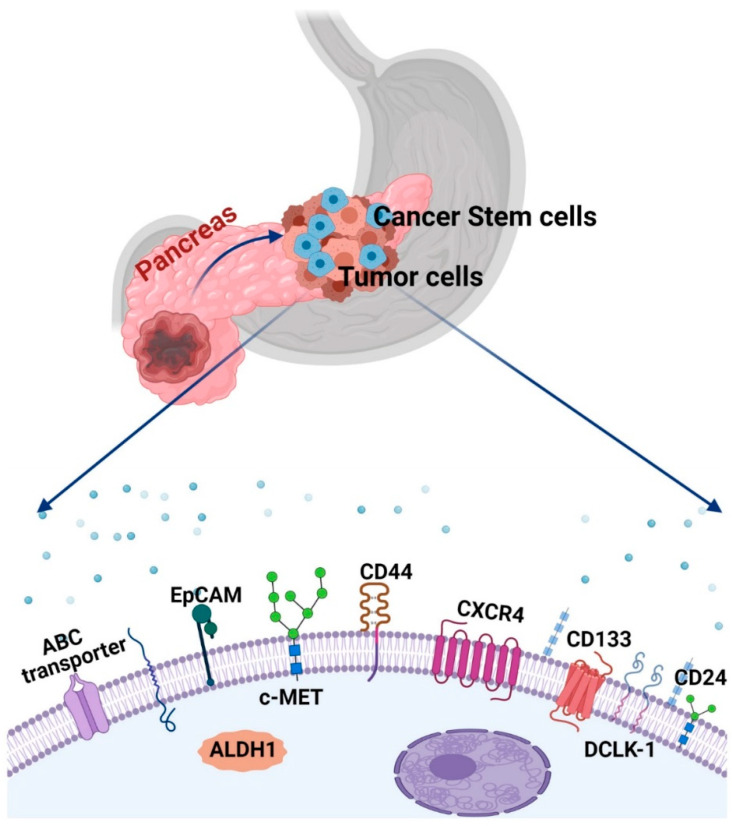
A schematic representation of distinct cancer stem cell populations in pancreatic cancer (PC). ATP-binding cassette (ABC); Epithelial cell adhesion molecule (EpCAM); Aldehyde dehydrogenase 1 (ALDH1); C-X-C Motif Chemokine Receptor 4 (CXCR4); Doublecortin-like kinase 1 (Dclk1).

The PaCSCs were first identified by Li and colleagues in 2007 and represented less than 1% of all pancreatic cancer cells [21]. In fact, Li et al. identified a subpopulation of pancreatic cancer cells with the specific cell surface markers CD44+CD24+ESA+ as pancreatic CSCs, which showed stem-cell-like properties of self-renewal, the ability to produce differentiated progeny and could recapitulate features of the original tumor [21]. However, later studies showed that PaCSCs could be identified by multiple markers, including CD34, CD44, CD133, ESA, ALDH1, CXCR4, DCLK-1 and cMet (Figure 1) [22,23,24,25,26,27,28,29,30]. In this regard, CD24+/CD44+/EpCAM+ expression also coincided with poorly differentiated cells and was associated with a high proliferative perspective in the clinical assessment of PC [31,32]. Studies have now also proposed CD133, CXCR4, and cMet as PaCSCs specific cell markers and demonstrated that cMet positive cells from human pancreatic cancer have increased tumor potential and self-renewal ability [25,28,33,34,35,36]. In addition to the specific surface markers, PaCSCs also have high ALDH1 activity, which is important for the early differentiation of the stem cells. High levels of ALDH1 can provide chemotherapy protection and can be a potential target for the chemoresistance challenge [27,37]. Studies have also demonstrated that CD133+ cells in primary PC and PC cell lines have increased proliferative ability, which is a distinguishing characteristic of CSC. It is also shown that the subpopulation of CD44+CD24+ EpCAM+ cells strongly overlaps with the CD133+ population [38]. Of note, CD133+ cells were characterized as gemcitabine-resistant and essential for developing metastasis [39,40], thus an important marker for therapy [41].

Furthermore, CSCs exist within the tumor microenvironment (TME) along with other cellular components [42,43]. Therefore, understanding the interplay between CSCs and the TME is crucial. PDAC TME includes various types of collagen fibers like cancer-associated fibroblasts (CAFs), collagen type V, pancreatic stellate cells, mesenchymal stem cells, and immune cells [44]. CAFs have been recognized as an essential component of the TME which endure metabolic reprogramming within the CSC niche. CAFs also derive energy from the activation of autophagic programs to retain their enhanced proliferative and migratory capacity, as well as their successful secretion of cytokines and growth factors. CSCs have also demonstrated a high autophagic flux, thus playing a significant role in the resistance to CSCs therapy [45,46]. CSCs adjust their metabolism to their microenvironment by acquiring intermediate metabolic phenotypes or shifting from oxidative phosphorylation to glycolysis/Warburg effect [47,48].

### 1.2. Cancer Stem Cells and PC Heterogeneity

Malignant tumors comprise a heterogeneous population of cells representing several cellular and oncogenic states. Heterogeneous histology is found between tumors, as well as within tumors. More recent molecular studies have further established that the intertumor heterogeneity is greater than the intratumor heterogeneity during the initial stage of pancreatic metastasis [49]. In this regard, persistent genetic mutations are the hallmark of pancreatic cancer, and mutations in KRAS occur in most PDACs [50,51,52]. Additional mutations in other driver genes, such as p53, CDKN24, and Smad4/DPC4, may be attributed in the later stage of PDAC [53,54,55]. The phenotypic and functional diversification of CSCs can be motivated by the intra-patient genetic heterogeneity [56]. Therefore, the functional properties of various CSCs derived from human tumors with different genotypes would need to be determined to understand inter-patient diversity. However, in many cancers, including PDAC, certain genetic alterations may accrue in a sequence during disease progression [57,58]. Therefore, it is likely that different CSCs are associated with relapses and disease progression over the course of the disease advancement.

In 2015, Maddipati and Stanger used multicolor lineage tracing technology in a murine model of PDAC to follow the cellular dynamics of in vivo metastasis. They investigated the heterogeneity of primary tumors from the preliminary stages of tumor growth to developing metastasis. Interestingly, they found that at secondary sites, only a small subset of cells could successfully seed and proliferate, though metastasis would start as a polyclonal population. This study supported the perception of clonal diversity and evolution of the CSC-like cells for driving the formation of metastatic tumors in secondary organs in PDAC patients [59]. Furthermore, the patient-derived PDAC tumors were demonstrated to be functionally distinct in serial xenografts and could be traced to a few cells, the tumor-initiating cells (TICs) with high plasticity. These studies emphasize the importance of developing efficient TIC-directed therapies for human PDAC [60,61]. Hence, far, it is not completely clear how genetic evolution and diversification impact developing distinct CSCs, or how CSCs affect the clonal composition of tumors. Therefore, a systematic examination of genetic mutations in CSC heterogenicity, their phenotypes, and functional properties may address these queries.

### 1.3. Strategies for Identification and Isolation of PaCSCs

PaCSCs identification and characteristics will be beneficial in providing earlier diagnosis and more successful treatments for PC patients. Despite the advances in CSC biology, these attributes are not completely understood. Owing to the heterogeneity in CSCs and technical difficulties in the CSC population isolation, multiple methodologies have been applied to enrich the isolation of exclusive CSC populations from heterogeneous cancer cells in pancreas tumor mass. Currently, these strategies used are limited to using the cell surface markers (e.g., EpCAM, CD133, CD44, CXCR4, ABCG2, CCR7, Oct4, Sox2, and Nanog), functional assays (spheroid formation, colony formation, ALDHs activity, SP assay), aldefluorine assay and Hoechst-33,342 dye method (Table 1) [22,27,62,63]. As noted before, the presence of cell surface markers CD44+/CD24+/EpCAM+ or CD133 for CSCs in PC was first shown by Li et al. and Hermann et al. [21,64]. In both cases, they demonstrated that these markers could distinguish PaCSCs from normal cells, which have self-renewing and multipotential capabilities. It was also demonstrated that CD133+/CXCR4+ CSCs were accountable for a metastatic phenotype of the pancreatic tumors. Thus, CD133, EpCAM, CD44, and CXCR4 are commonly used to isolate and examine the involvement of the CSC population in pancreatic cancer progression [33,65,66,67] and constitute prime targets for the treatment.

PDAC tumors have also been shown to contain a subpopulation of cells with distinct auto-fluorescent intracellular vesicles. These auto-fluorescent vesicles can, therefore, be used to classify and separate subsets of cells with robust CSC properties, including improved self-renewal, pluripotency-associated gene expression, and significant chemoresistance [68]. In this discovery, the author found that these fluorescent vesicles accumulate riboflavin and express the ATP-binding cassette (ABC) transporter ABCG2. Riboflavin is a spontaneous substrate for ABCG2, which is expressed on the surface of many cancer cells that primarily acts to reduce the intracellular concentration of chemotherapeutic drugs [69]. In PaCSCs, ABCG2 is overexpressed [68]. ER-derived cytoplasmic ABCG2-coated vesicles can serve as riboflavin intracellular sinks, resulting in the formation of the auto-fluorescent vesicle of the CSC. ABCG2-generated auto-fluorescent marker development is a significant advancement in the context of both identification of PaCSCs and their molecular analysis [68]. Overall, there has been progressing in this field. However, no single marker or in combination may classify all the CSC subpopulations present within a tumor. Significant research in the future should be dedicated to discovering new markers that can specifically fine-tune the ability to differentiate all CSC populations in the pancreatic tumor microenvironment.

## 2. Targeting Major Signaling Pathways to Regulate CSCs for PC Therapy

**Figure 2:**
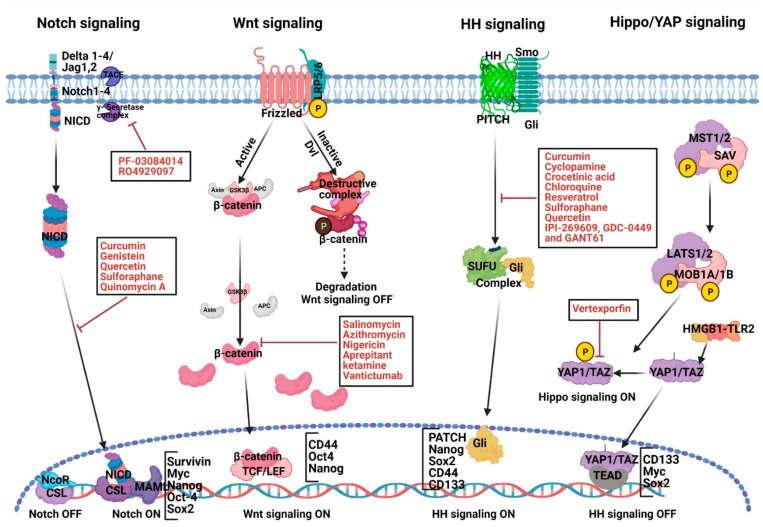
A schematic representation of major signaling cascades in normal stem cells vs. pancreatic cancer stem cells and their therapeutic strategies. Jagged1,2 (JAG1,2); Notch intracellular domain (NICD); Low-density lipoprotein receptor-related protein 5/6 (LRP5/6); Dishevelled (Dvl); Adenomatous polyposis coli (APC); T-cell factor/lymphoid enhancer factor (TCF/LEF); Hedgehog (Hh).

It is widely accepted that chemotherapeutic outcomes can be improved greatly by targeting particular signaling pathways important for the regulation of PaCSCs. Multiple signaling pathways are altered in PaCSCs, including Notch, Wnt, hedgehog, Hippo, AKT/mTOR, MAPK-ERK, and Nodal/activin signaling (Figure 2). Specifically, Notch, Wnt, and hedgehog have been of unique importance in PaCSCs, due to their significant role in pancreatic embryonic development and differentiation [70,71,72]. These signaling pathways also play an important role in the self-renewal of PaCSCs, tumor development, invasion, metastasis, and therapy resistance. Understanding the causal significance of these cellular pathways in CSCs development and progression will facilitate developing new therapeutic approaches to treat this dismal disease.

### 2.1. Abnormal Notch Signaling Activation and Therapeutic Strategies in PC Development

The Notch-signaling is a constitutive pathway, which acts as an essential regulator in the differentiation and self-renewal process of normal pancreas development. Notch signaling also regulates cell proliferation, cell survival, apoptosis, and cancer development in PC [73]. Notch-signaling plays a considerable role in stem cell differentiation, and aberrant Notch activation is associated with developing PC [74]. The mammalian Notch receptor family (Notch 1–4) gets activated through a sequence of cleavage events, which results in the release of the intracellular domain of Notch, which translocates to the nucleus. It then stimulates the expression of various target genes like survivin, cMyc, Nanog, Oct-4, and Sox2, which are important for CSC self-renewal. Studies have also observed that PaCSCs exhibit significantly higher levels of Notch expression [75,76,77,78] and thus can be a promising therapeutic approach in pancreatic cancer development.

Some of the Notch inhibitors that have been employed in PC therapy in recent years are summarized here: Notch signaling is triggered by receptor cleavage through γ-secretase activity. It has been observed that γ-secretase inhibitors, PF-03084014 and MRK-003, triggers apoptosis and interfere with cell proliferation in several human cancer, including PC [77,79]. Moreover, PF-03084014 alone or in combination with gemcitabine remarkably inhibited intracellular activation of the Notch and subsequent transcriptional regulation of *Hes-1* and *Hey-1* (Notch targets), inducing 75% tumor regression in the xenograft model for pancreatic cancer. PF-03084014, when used in combination with gemcitabine, also inhibited PaCSCs significantly [77]. Recent studies have also shown that “natural agents”, including curcumin, genistein, quercetin, and sulforaphane, are capable of inhibiting Notch expression, thus can be a promising therapeutic agent to target PaCSCs. Curcumin (diferuloylmethane) is an active compound found in *Curcuma longa*, which is commonly used in food as a flavoring agent and has been shown in preclinical studies to have antitumor activity against many forms of cancer, including PC [80,81]. Curcumin was recently shown to inhibit the sphere-forming capacity (pancreatosphere) of PC cells and attenuation of CD44 and EpCAM PaCSCs markers in gemcitabine-resistant PC cells, which contain a high proportion of CSCs [82]. Genistein, quercetin and flavonoids are also shown to play a causal role in diminishing the Notch signaling induced PaCSCs [75,83,84]. Sulforaphane is another natural compound, which has potential specificity in regulating pancreatic tumor-inducing cells [85]. Further, quercetin and sulforaphane exhibited a synergistic effect in targeting PaCSCs [86,87]. Sulforaphane also improved cell sensitivity to numerous chemotherapeutic agents like cisplatin, gemcitabine, doxorubicin, and 5-fluorouracil, particularly by targeting the Notch-1 signaling induced CSC in PC [88]. Overall, it is noteworthy that targeting the Notch1 signaling pathway by employing natural agents alone or in combination with conventional chemotherapeutic drugs, which specifically inhibit PaCSCs growth, could be a safer strategy to obtain a better treatment outcome for patients diagnosed with PC.

### 2.2. Abnormal Wnt Signaling Activation and Therapeutic Strategies in PC Development

The canonical Wnt-signaling pathway is a crucial evolutionarily conserved pathway for embryonic development and tissue homeostasis. In the absence of Wnt ligand, the cytoplasmic β-catenin is phosphorylated for proteasome-dependent degradation by a “destruction complex” consisting of axin, protein phosphatase 2A (PP2A), adenomatous polyposis coli (APC), glycogen synthase kinase 3β (GSK3β), and casein kinase I𝛼 (CKI𝛼) [89]. In several human cancers, including PC, aberrant Wnt/β-catenin signaling is one of the major drivers for cancer progression [90]. Active Wnt/-catenin has been shown to be involved in approximately 65% of pancreatic adenocarcinomas, though mutations of the β-catenin (CTNNB1) [91]. Several studies have shown that targeting the Wnt/β-catenin signaling pathway enhances the sensitivity of PC to chemotherapeutic agents by targeting PaCSCs subsets [92]. Current clinical trials using Wnt-signaling inhibitors have shown promising results, though, at present, there are no FDA-approved specific Wnt-inhibitors available for clinical usage. Salinomycin is an antibacterial and coccidiostat ionophore drug, which is shown to reduce tumor development and metastasis of PC [93]. Salinomycin was able to increase the cytotoxic effects of traditional therapy of gemcitabine in PaCSCs, by interfering with Wnt signaling [94]. Another FDA-approved antibiotic, azithromycin, has also been shown to inhibit the tumorsphere development in PC [95]. Tigecycline, a relatively new antibiotic produced in response to antibiotic resistance, also has been proven to minimize the formation of CSCs in the pancreas, breast, lung, and prostate [96,97,98,99]. Ketamine, which is an anesthetic and antidepressant, also reduced CSCs traits and tumor growth in CRC and PC cancer by targeting Wnt activity [100,101]. Vantictumab, a monoclonal antibody, which inhibits Wnt signaling by targeting the FZD receptor, showed promising outcomes in combination with gemcitabine and could halt the metastasis of pancreatic ductal epithelial (HPDE) and HPDE/KRAS cells [102]. Three-phase 1b clinical trials of vantictumab in combination with nab-paclitaxel and gemcitabine have been conducted, where it was well tolerated in PC patients except for slight bone toxicity [103].

### 2.3. Abnormal HH Signaling Activation, PaCSCs and Therapeutic Strategies

Hedgehog (HH) signaling plays a critical role in several biological processes, including embryo development, organogenesis, and tissue rejuvenation [104,105]. Hedgehog signaling can play a dual role and may function as mitogen or differentiation. Three hedgehog homologs have been studied in mammals: sonic hedgehog (sHH), Indian hedgehog (iHH), and desert hedgehog (dHH). These hedgehog ligands bind its 12-pass transmembrane receptor, patched (PTCH1), which leads to internalization, degradation, and release of signal transducer Smoothened (SMO), a G-protein coupled receptor (GPCR) and subsequent dissociation of the suppressor of fused (SUFU)–Gli1 complex. Almost 70% of PC patients had increased HH signaling [106]. Moreover, it has been shown that silencing of a single Gli1 allele ensued distinctive inflammatory response and inappropriate PC-associated stroma restoration in in vivo PC model [107]. The HH pathway also regulates the maintenance of somatic stem cells and pluripotent cells, and this pathway may be correlated with the maintenance potentiality of CSCs [108]. Li et al. have recently verified that the HH pathway is involved in the tumorsphere formation in PC cells [109]. Inhibition of HH signaling by Smo knockdown blocks PaCSCs’ self-renewal, EMT, invasion, chemoresistance, and tumorigenesis [110]. Therefore, targeting CSCs through the HH signaling pathway may develop the clinical advantage for PC patients.

Numerous HH pathway inhibitors have been identified in the past years. Cyclopamine, a natural compound, was the first to be recognized to interfere with the HH pathway. It inhibited the Smo activation, sHH target [111]. Treatment with cyclopamine also reduced Gli1 expression in PC cells [112,113]. Moreover, it downregulated CD44 and CD133 expression in gemcitabine-resistant PC cells and induced gemcitabine sensitivity [114]. Chloroquine (CQ), the antimalarial agent, significantly reduced pancreatic CSCs by inhibiting the HH-signaling pathway. Further, the combination of CQ with gemcitabine synergistically reduced the overall survival of PDAC-derived xenografts [115]. Saffron-isolated crocetinic acid, yet another compound, also inhibits PaCSCs by targeting the self-renewal potency through interference in hedgehog signaling [116]. Curcumin and Sulforaphane also inhibited the self-renewal of PaCSCs by downregulating sHH signaling [117,118,119]. In addition, two other small-molecule inhibitors of the HH signaling pathway, IPI-269609, and GDC-0449, were tested [120,121]. A pilot study was performed on 25 PDAC patients using GDC-0449 in combination with gemcitabine. The treatment inhibited HH signaling without significant changes in PaCSCs. In addition, GDC-0449 and gemcitabine combinational therapy failed to be superior to gemcitabine alone in the advanced PC treatment [122].

### 2.4. Targeting Hippo Signaling

The hippo-signaling pathway, reviewed in detail in other places, plays a key role in the preservation of tissue homeostasis, organ size, and tumorigenesis [123,124]. In brief, when the hippo-signaling component, LATS1/2, is inactive, it activated YAP/TAZ transfers to the nucleus, which induces gene transcription through interaction with transcription factors, like the TEAD family. An alteration in this pathway is often involved in most types of cancer development, including PC [125]. YAP1/TAZ and TEAD are often upregulated in PC tumor cells. TAZ has been shown to induce EMT and facilitate PC progression and development. The Hippo signaling protein YAP acts on multiple aspects of PDAC. In addition, increased inflammation and severely suppressed immune response are typical features of PC. Studies have proven that YAP stimulates the differentiation and accumulation of myeloid-derived suppressor cells (MDSCs), which contribute to a strong immunosuppressive microenvironment in PDAC. Recent research has shown that YAP is downstream of HMGB1-TLR2 signaling and possesses the ability to enhance PC stemness by inducing CD133 expression [126]. YAP overexpression in PDAC also promotes cancer metastasis and chemoresistance [127,128]. Taken together, the above studies suggest that the Hippo signaling protein YAP/TAZ can be a crucial target to control pancreatic cancer. Of note, YAP1 and TAZ can regulate the direct activation of the JAK-STAT3 signaling pathway to regulate pancreatic cancer in mouse models [129]. YAP has also shown to be a critical oncogenic KRAS effector and a promising therapeutic target for pancreatic cancer [130,131]. Overall, the Hippo signaling proteins YAP and TAZ are important targets for the prevention and treatment of pancreatic cancer.

### 2.5. Targeting JAK-STAT Pathway

The Janus kinase (JAK) and signal transducer and activator of transcription (STAT) pathways are involved in different cellular functions, including cell proliferation, angiogenesis, metastasis, and immune evasion, cytokines, and growth factors signaling pathways. JAK-STAT signaling pathways are upregulated in various cancers, including PC [132,133]. STAT3 is constitutively activated in oncogenic KRAS-driven PC [134]. Modulation of the JAK/STAT pathway in CSCs has been shown to enhance the expansion potentiality of cancer-forming cells. Studies have shown that gemcitabine treatment increases the ratio of CD24+ and CD133+ cells to increase stemness in PC by increasing the Nox/ROS/NF*κ*B/STAT3 signaling cascade [135]. Stromal-derived IL-6/Jak2/STAT3 signaling plays a major role in PaCSCs functioning and PC progression [136]. In addition, the combination of the Notch inhibitor GSI-IX and the JAK2/STAT3 inhibitor AG-490 has been proven as a promising PC therapeutic agent [137]. The combination treatment of indole-3-carbinol (I3C) with genistein substantially inhibits constitutive activated STAT3 expression in PC cells [138]. The survivin/BIRC5 gene expression is downregulated by curcumin and inhibits constitutive STAT3 phosphorylation in human PC [139]. Similarly, resveratrol also inhibits phosphorylation of STAT3 in in vitro PC cells [140].

### 2.6. Targeting PI3K/Akt/mTOR Signaling

For several physiological and pathological conditions, PI3K/Akt and mTOR signaling pathways are important, such as cell proliferation, angiogenesis, metabolism, differentiation, and survival. It is thought that PI3K/Akt/mTOR signaling pathway is aberrantly reactivated in PaCSCs [141]. A recent study proved that inhibition of the PI3K/Akt/mTOR pathways resulted in diminished stem cell characteristics of PC and tumor advancement. Sharma et al. further demonstrated proficiency in combining therapy of PI3K/mTOR inhibitor (NVP-LDE-225) and sHH inhibitor (NVP-BEZ-235) on PaCSCs characteristics, microRNA regulation network, and tumor growth by controlling the expression of pluripotency conserving factors Nanog, Oct-4, Sox-2, and cMyc along with repression of Gli transcription. Interaction between these drugs has also been shown on PaCSCs using Pan^kras/p53^ mice [141]. The suppression of the AKT-activation was also demonstrated by γ-tocotrienol, resulting in the downregulation of p-GSK-3β along with nuclear translocation of FoxO3. Vitamin E δ-tocotrienol has also been shown to induce apoptosis and inhibit cell survival and proliferative pathways, such as PI3-kinase/AKT and ERK/MAP kinases, partly by curbing the expression of Her2/ErbB2 [142,143].

### 2.7. Targeting MAPK-ERK Pathway

The MAPK pathway plays a crucial role in controlling a wide variety of cellular signals, which regulate cell growth and differentiation. In PC, KRAS transduces MAPK signaling to regulate cell proliferation, differentiation, and apoptosis. KRAS mutations activate downstream signaling pathways, such as extracellular signal-regulated kinase (ERK), subsequently resulting in cell transformation and tumorigenesis [144]. In contrast to CD133- cells, MAPK signaling activation results in resistance to TGF-β-induced apoptosis in CD133+ cells. Additionally, CD133+ CSCs specifically show increased phosphorylation of ERK1/2 due to increased MAPK signaling [145]. Moreover, the chemokines CCL21/CCR7 signaling induces EMT and ERK/NF*κ*B pathways to promote PC cell metastasis [146]. Chai et al. found a correlation between the phosphorylation of P70S6K and ERK1/2. They also identified metformin as a potent therapeutic agent to inhibit this activation, which in turn inhibited PC cell proliferation [147]. Therefore, metformin could be a persuasive agent to inhibit the MAPK signaling from controlling PaCSCs proliferation.

### 2.8. Targeting CXCR4 Signaling

Stromal cell-derived factor 1: SDF-1 and its G-protein-coupled receptor CXCR4 have been shown to play a crucial role in PC metastasis [148]. SDF1 is a small pro-inflammatory cytokine that stimulates angiogenesis. CXCR4 is a receptor for SDF-1. After SDF-1 binds to CXCR4, it initiates multiple signal transduction pathways, including the ERK-2, PI3K, MAPK, and NF*κ*B, thus regulating cell survival, proliferation, and chemotaxis [109,149]. Since CXCR4-expressing tumor cells can migrate to normal tissue expressing SDF-1, the SDF-1/CXCR4 pathway has been shown to play a crucial role in mediating tumor metastasis [149]. More recently, Hermann et al. showed that CD133+CXCR4+ pancreatic cancer cells have high metastatic potential and depletion of these CD133+CXCR4+ cells abrogated the metastatic potential. They also discovered that PC patients with metastasis of the lymph node had greater numbers of CD133+CXCR4+ migrating PaCSCs. These results signify that the SDF-1/CXCR4 axis is important in PaCSCs and metastasis, and modifying this axis may have clinical applications in alleviating PC [64,150]. Recent research also has revealed that blocking receptors targeting anti-CXCR4 and small-molecule inhibitor AMD3100 inhibited the migration and metastasis efficacy of PaCSCs in a mouse model [64]. Chloroquine also inhibited PaCSCs survival via blocking of CXCL12/CXCR4 signaling [115].

### 2.9. Targeting NODAL/ACTIVIN Signaling

Nodal and activin, part of the TGF-β superfamily, has also been shown to play a significant role in maintaining embryonic stem cell pluripotency and differentiation during embryogenesis. Nodal and activin exert their biological role by binding type I (Alk4 and Alk7) or II (ActRIIA and ActRIIB) receptors to the cell surface, resulting in the phosphorylation of SMAD proteins resulting in the activation of target gene expression, including Nanog and Oct4. Activin A has been reported to be able to sustain stemness in human embryonic stem cells by inducing Oct4, Nanog, Wnt3, FGF and inhibiting the BMP signal [151]. Lonardo et al. have recently stated that Nodal and activin are highly expressed in PaCSCs and control the ability of CSC to self-renew. In addition, they found that inhibition of the Alk4/7 nodal/activin receptor in PaCSCs abolished their self-renewal ability, tumorgenicity, and resistance to gemcitabine [152]. Thus, inhibition of Nodal signaling reduced tumorigenicity of PaCSCs, signifying that Nodal could be a potential target for PC therapy development.

Overall, our extensive insight on the signaling pathways involved in PaCSCs functioning described above is summarized in Table 2. Compounds or combination therapies that simultaneously target multiple signaling pathways in PaCSCs progression can yield better results than single-target treatments in controlling pancreatic tumor frequency, recurrence, and drug resistance.

### 2.10. Targeting MicroRNAs to Regulate CSCs for PC Therapy

Over the last decade, there has been increasing evidence suggesting that the maintenance of pluripotency in PaCSCs is governed not solely by different signaling cascades but also by core genetic regulators. MicroRNAs (miRNAs) have been established to act as pivotal regulators of the post-transcriptional regulation of gene expression. The modified expressions of miRNAs are correlated with poor clinical outcomes of PC patients [154,155,156]. Emerging evidence firmly indicates that miRNAs play a crucial role in tumor growth and development. Differential expression of certain miRNA in PaCSCs has recently been investigated [157]. Singh et al. documented that PC recur due to small but distinct CSC populations, which are in turn regulated by miRNAs [158]. The study identified a series of miRNAs in gemcitabine-resistant MIAPaCa-2 cancer cells and in clinical metastatic pancreatic cancer tissues where they were either upregulated (e.g., miR-146) or downregulated (e.g., miRNA-205, miRNA-7). Transfection of miR-205 decreased ALDH-positive CSC potentials, which resulted in gemcitabine chemosensitivity restoration in the gemcitabine-resistant MIAPaCa-2 cell line [158]. Another study by Bao et al. stated that miR-26a plays an important role in controlling the expression of EZH2 and EpCAM in PC cells, and re-expression of let-7b, miR-26a, and miR-200b decreases the ability of MIAPaCa-2 cancer cells to form pancreatospheres [159]. These findings provide new avenues for the treatment and detection of PC. A brief summary of differentially expressed miRNAs with their targets and implications in pancreatic cancer is tabulated in Table 3.

## 3. Conclusions

The discovery of CSCs has introduced a number of interesting and important concepts and has revealed the relevance of a small subset of cells that could sustain the tumor. These cells are most resilient in tumors and thus can reestablish tumors after treatment. Although further studies are needed, the new developments in targeting PaCSCs are expected to have a high impact on the treatment of PC, the mechanisms for regulating clinical chemoresistance and the potential for developing novel therapies in coming years.

The most effective way of targeting pancreatic cancer is by destroying the CSC niche or by altering the expression of the important players, which support the survival of CSCs. Although several methods have been employed to isolate CSCs, there are still limitations with current methods. Different markers have been established in PC. However, they are also important for other cancers. Therefore, more specific markers to identify PaCSCs are urgently needed. Further, the signaling pathways, such as Notch, Wnt and Shh, are also altered in CSCs. Advances in genetic engineering, tumor metabolism, and the microenvironment could provide a framework to better understand some of these mechanism/s and identify novel therapeutic targets. Future clinical trials should focus on novel therapeutic agents that target CSCs and the important molecules in the signaling pathways to control the aggressiveness of pancreatic cancer. Effective treatment of pancreatic cancer may require the administration of conventional chemotherapeutic agents along with reagents that deplete PaCSCs. Eventually, understanding distinct facets of PaCSCs biology might provide the foundation for clinical trials with a positive impact on new and successful therapies to combat anticancer therapy resistance in pancreatic cancer.

## Figures and Tables

**Table 1:** Approaches for cancer stem cells (CSCs) detection and isolation.

S. No.	Detection Technology	Advantages	Drawbacks and Limitations
1.	Fluorescence-activated cell sorting (FACS)	Highly flexible technique with a large range of stem cell sorting capabilities Very precise Multiparameter isolation	Complicated method Viability of recovered cells is low High cost Time-consuming There is no universal marker for identifying CSCs Require cells in suspension, and in this state, cells clump together, and metabolism may be altered
2.	Magnetic-activated cell sorting (MACS)	Fast and easy method in the isolation of CSCs with the capability of isolating small populations of the cells within the tumor bulk High specificity	Monoparameter separation Involves a cell suspension solution rather than a solid sample There is no universal marker for identifying CSCs
3.	Aldehyde dehydrogenase 1 (ALDH1) activity	Stability than the cell surface markers ALDH1-positive cells displayed increased sphere formation capability, self-renewal properties, tumorigenicity and high expression of stemness genes	Low specificity (It can be used either for the normal or CSC)ALDH1 may not be a proper CSC marker for all tumor types
4.	Spheroid formation assay	Simple assay There is no need for expensive laboratory facilities	Heterogeneity and presence of differentiated cells In spheroid formation, there is no quiescent CSCs
5.	Colony formation	Simple and easy	Freshly prepared required To ensure that each colony results from a single cell, proper cell dilution is needed
6.	SP assays	Easier and reliable method Promising method for identifying stem cell and progenitor populations in different tissues and numerous cancers There are no unique cellular markers needed for CSC isolation	Lack of homogeneity in the SP staining protocols Unspecified method for SP population in various tumors Low specificity Lack of purity Toxicity of Hoechst 33342

**Table 2:** Summary of therapeutic agents targeting different signaling and PaCSCs.

S. No.	Signaling Pathway	Therapeutic Agents (Function)/Small Molecule Compounds	References
1.	Notch	Curcumin (diferuloylmethane), genistein (soy isoflavonoid), quercetin (polyphenol and flavonoid), sulforaphane (phytochemical), PF-03084014 (γ-secretase inhibitor), MRK-003 (γ-secretase inhibitor)	[77,78,79,80,81,82,83,84,85,86,87,88]
2.	Wnt, EMT	Salinomycin, azithromycin, tigecycline, and ketamine (anesthetic and antidepressant), vantictumab (monoclonal antibody)	[93,94,95,96,97,98,99,100,101,102,103]
3.	Hedgehog	Curcumin (diferuloylmethane), cyclopamine (phytochemical), crocetinic acid, chloroquine (antimalarial agent), sulforaphane (phytochemical), quercetin (polyphenol and flavonoid), IPI-269609, and GDC-0449	[111,112,113,114,115,116,117,118,119,120,121,122]
4.	Hippo-signaling	Verteporfin (porphyrin molecule)	[153]
5.	JAK-STAT pathway	AG-490, curcumin (diferuloylmethane), resveratrol (polyphenol), indole-3-carbinol (I3C) and genistein	[137,138,139,140]
6.	PI3K/Akt/mTOR-signaling	Rapamycin, AZD8055, NVP-LDE-225, NVP-LDE-225, NVP-BEZ-235, δ-tocotrienol (vitamin E)	[141,142,143]
7.	MAPK-ERK pathway	Metformin	[147]
8.	CXCR4-signaling	AMD3100 (small-molecule inhibitor), chloroquine (antimalarial agent)	[64]
9.	NODAL/ACTIVIN-signaling	SB431542	[152]

**Table 3:** Summary of differentially expressed microRNAs and their targets in pancreatic cancer.

miRNA/s	Sample Type/Site of Action	Regulation	Target (−ve)/(+ve)	Implication	Reference
miR-146	Metastatic pancreatic cancer tissues vs. normal control	Up			[157]
miRNA-205, miRNA-7		Down			
miR-26a, miR-200b	PDAC samples vs. normal control			EZH2, EpCAM, pancreatospheres	[158]
miR-21, miR-27a, miR-146a, miR200a and miR-196a	Pancreatic cancer tissue vs. paraneoplastic normal pancreatic tissues	Up		51	[160]
miR-217, miR-20a, and miR-96		Down		107	
miR-198, miR-650,	Pancreatic adenocarcinomas and chronic pancreatitis vs. normal pancreas	Up		43	[161]
miR-130b, miR-141, miR-194 and miR-219-1-3p		Down			
				41	
miR-21-5p, -23a-3p, -31-5p, -34c-5p, -93-3p, -135b-3p, -155-5p, -186-5p, -196b-5p, -203, -205-5p, -210, -222-3p, -451, -492, -614, and miR-622	Pancreatic cancer vs. healthy control	Up		17	[162]
miR-122-5p, -130b-3p, -216b, -217, and miR-375		Down		5	
miR-21, miR-155, miR-210, miR-221, and miR-222	PDAC vs. healthy control	Up		5	[163]
miR-31, miR-122, miR-145, and miR-146a		Down		4	
miR-18a	Plasma of pancreatic cancer patient vs. healthy control	Up			[164]
miR-21	Plasma of pancreatic cancer patient vs. healthy control	Up		54	[165]
miR-146a		Down		37	
miR-143	Metastatic pancreatic cancer	Down	GEF1, GEF2, K-RAS, MMP-2, and MMP-9 (−ve)	Metastasis, invasive potential ↑, EMT ↑	[166]
miR-126	PDAC progressive samples with metastasis	Down	ADAM9 (−ve)	Metastasis, invasive potential ↑, EMT ↑	[167]
miR-146a	Pancreatic cancer vs. normal human pancreatic duct	Down	EGFR, MTA-2, IRAK-1, NF*k*B (−ve)	Invasive potential ↑	[168]
miR-218	Metastatic pancreatic cancer; microarray analysis/pancreatic cancer sample	Down	ROBO1 ↑	Progression and lymphatic metastasis ↑, Invasion and migration potential ↑	[169,170]
miR-4295	PDAC cells	Up	GPC5 ↓	Proliferation, invasion and Wnt/β-catenin signaling ↑	[171]

## Data Availability

No new data were created or analyzed in this study. Data sharing is not applicable to this article.

## References

[1] Global Cancer Observatory. https://gco.iarc.fr/.

[2] Survival Rates for Pancreatic Cancer. https://www.cancer.org/cancer/pancreatic-cancer/detection-diagnosis-staging/survival-rates.html.

[3] Khalaf N., El-Serag H.B., Abrams H.R., Thrift A.P. (2020). Burden of Pancreatic Cancer: From Epidemiology to Practice. Clin. Gastroenterol. Hepatol..

[4] Huang J., Lok V., Ngai C.H., Zhang L., Yuan J., Lao X.Q., Ng K., Chong C., Zheng Z.-J., Wong M.C.S. (2021). Worldwide Burden of, Risk Factors for, and Trends in Pancreatic Cancer. Gastroenterology.

[5] Bailey P., Chang D.K., Nones K., Johns A.L., Patch A.-M., Gingras M.-C., Miller D.K., Christ A.N., Bruxner T.J.C., Quinn M.C. (2016). Genomic Analyses Identify Molecular Subtypes of Pancreatic Cancer. Nature.

[6] Moffitt R.A., Marayati R., Flate E.L., Volmar K.E., Loeza S.G.H., Hoadley K.A., Rashid N.U., Williams L.A., Eaton S.C., Chung A.H. (2015). Virtual Microdissection Identifies Distinct Tumor- and Stroma-Specific Subtypes of Pancreatic Ductal Adenocarcinoma. Nat. Genet..

[7] Korc M. (1998). Role of Growth Factors in Pancreatic Cancer. Surg. Oncol. Clin. N. Am..

[8] Neoptolemos J.P., Stocken D.D., Friess H., Bassi C., Dunn J.A., Hickey H., Beger H., Fernandez-Cruz L., Dervenis C., Lacaine F. (2004). A Randomized Trial of Chemoradiotherapy and Chemotherapy after Resection of Pancreatic Cancer. N. Engl. J. Med..

[9] Yue Q., Gao G., Zou G., Yu H., Zheng X. (2017). Natural Products as Adjunctive Treatment for Pancreatic Cancer: Recent Trends and Advancements. Biomed. Res. Int..

[10] Zong Y., Peng Z., Wang X., Lu M., Shen L., Zhou J. (2020). Efficacy and Safety of Nab-Paclitaxel Plus S-1 versus Nab-Paclitaxel Plus Gemcitabine for First-Line Chemotherapy in Advanced Pancreatic Ductal Adenocarcinoma. Cancer Manag. Res..

[11] Mortezaee K. (2021). Enriched Cancer Stem Cells, Dense Stroma, and Cold Immunity: Interrelated Events in Pancreatic Cancer. J. Biochem. Mol. Toxicol. N/A.

[12] Das P.K., Pillai S., Md Rakib A., Khanam J.A., Gopalan V., Lam A.K.Y., Islam F. (2020). Plasticity of Cancer Stem Cell: Origin and Role in Disease Progression and Therapy Resistance. Stem Cell Rev. Rep..

[13] Garcia-Mayea Y., Mir C., Masson F., Paciucci R., LLeonart M.E. (2020). Insights into New Mechanisms and Models of Cancer Stem Cell Multidrug Resistance. Semin. Cancer Biol..

[14] Marcu L.G. (2020). Cancer stem cells as therapeutic targets of pancreatic cancer. Fundam. Clin. Pharmacol..

[15] Valle S., Martin-Hijano L., Alcalá S., Alonso-Nocelo M., Sainz B. (2018). The ever-evolving concept of the cancer stem cell in pancreatic cancer. Cancers.

[16] Tang D. (2012). Understanding cancer stem cell heterogeneity and plasticity. Cell Res..

[17] Eun K., Ham S.W., Kim H. (2017). Cancer stem cell heterogeneity: Origin and new perspectives on CSC targeting. BMB Rep..

[18] Lee C.J., Dosch J., Simeone D.M. (2008). Pancreatic Cancer Stem Cells. JCO.

[19] Nimmakayala R.K., Batra S.K., Ponnusamy M.P. (2019). Unraveling the Journey of Cancer Stem Cells from Origin to Metastasis. Biochim. Biophys. Acta (BBA) Rev. Cancer.

[20] Steinbichler T.B., Savic D., Dudás J., Kvitsaridze I., Skvortsov S., Riechelmann H., Skvortsova I.-I. (2020). Cancer Stem Cells and Their Unique Role in Metastatic Spread. Semin. Cancer Biol..

[21] Li C., Heidt D.G., Dalerba P., Burant C.F., Zhang L., Adsay V., Wicha M., Clarke M.F., Simeone D.M. (2007). Identification of Pancreatic Cancer Stem Cells. Cancer Res..

[22] Hsieh M.J., Chiu T.-J., Lin Y.C., Weng C.-C., Weng Y.-T., Hsiao C.-C., Cheng K. (2020). Inactivation of APC Induces CD34 Upregulation to Promote Epithelial-Mesenchymal Transition and Cancer Stem Cell Traits in Pancreatic Cancer. Int. J. Mol. Sci..

[23] Hong S.P., Wen J., Bang S., Park S., Song S.Y. (2009). CD44-Positive Cells Are Responsible for Gemcitabine Resistance in Pancreatic Cancer Cells. Int. J. Cancer.

[24] Li X., Zhao H., Gu J., Zheng L. (2015). Prognostic Value of Cancer Stem Cell Marker CD133 Expression in Pancreatic Ductal Adenocarcinoma (PDAC): A Systematic Review and Meta-Analysis. Int. J. Clin. Exp. Pathol..

[25] Nomura A., Banerjee S., Chugh R., Dudeja V., Yamamoto M., Vickers S.M., Saluja A.K. (2015). CD133 Initiates Tumors, Induces Epithelial-Mesenchymal Transition and Increases Metastasis in Pancreatic Cancer. Oncotarget.

[26] Wei H.-J., Yin T., Zhu Z., Shi P.-F., Tian Y., Wang C.-Y. (2011). Expression of CD44, CD24 and ESA in Pancreatic Adenocarcinoma Cell Lines Varies with Local Microenvironment. Hepatobiliary Pancreat. Dis. Int..

[27] Kahlert C., Bergmann F., Beck J., Welsch T., Mogler C., Herpel E., Dutta S., Niemietz T., Koch M., Weitz J. (2011). Low Expression of Aldehyde Deyhdrogenase 1A1 (ALDH1A1) Is a Prognostic Marker for Poor Survival in Pancreatic Cancer. BMC Cancer.

[28] Maréchal R., Demetter P., Nagy N., Berton A., Decaestecker C., Polus M., Closset J., Devière J., Salmon I., Van Laethem J.-L. (2009). High Expression of CXCR4 May Predict Poor Survival in Resected Pancreatic Adenocarcinoma. Br. J. Cancer.

[29] Bailey J.M., Alsina J., Rasheed Z.A., McAllister F.M., Fu Y., Plentz R., Zhang H., Pasricha P.J., Bardeesy N., Matsui W. (2014). DCLK1 Marks a Morphologically Distinct Subpopulation of Cells With Stem Cell Properties in Preinvasive Pancreatic Cancer. Gastroenterology.

[30] Ito H., Tanaka S., Akiyama Y., Shimada S., Adikrisna R., Matsumura S., Aihara A., Mitsunori Y., Ban D., Ochiai T. (2016). Dominant Expression of DCLK1 in Human Pancreatic Cancer Stem Cells Accelerates Tumor Invasion and Metastasis. PLoS ONE.

[31] Ohara Y., Oda T., Sugano M., Hashimoto S., Enomoto T., Yamada K., Akashi Y., Miyamoto R., Kobayashi A., Fukunaga K. (2013). Histological and Prognostic Importance of CD44+/CD24+/EpCAM+ Expression in Clinical Pancreatic Cancer. Cancer Sci..

[32] Zhu J., He J., Liu Y., Simeone D.M., Lubman D.M. (2012). Identification of Glycoprotein Markers for Pancreatic Cancer CD24+CD44+ Stem-like Cells Using Nano-LC–MS/MS and Tissue Microarray. J. Proteome Res..

[33] Cioffi M., D’Alterio C., Camerlingo R., Tirino V., Consales C., Riccio A., Ieranò C., Cecere S.C., Losito N.S., Greggi S. (2015). Identification of a Distinct Population of CD133 + CXCR4 + Cancer Stem Cells in Ovarian Cancer. Sci. Rep..

[34] Sleightholm R.L., Neilsen B.K., Li J., Steele M.M., Singh R.K., Hollingsworth M.A., Oupicky D. (2017). Emerging Roles of the CXCL12/CXCR4 Axis in Pancreatic Cancer Progression and Therapy. Pharmacol. Ther..

[35] Li C., Wu J., Hynes M., Dosch J., Sarkar B., Welling T.H., di Magliano M., Simeone D.M. (2011). C-Met Is a Marker of Pancreatic Cancer Stem Cells and Therapeutic Target. Gastroenterology.

[36] Herreros-Villanueva M., Zubia-Olascoaga A., Bujanda L. (2012). C-Met in Pancreatic Cancer Stem Cells: Therapeutic Implications. World J. Gastroenterol.

[37] Deng S., Yang X., Lassus H., Liang S., Kaur S., Ye Q., Li C., Wang L.-P., Roby K.F., Orsulic S. (2010). Distinct Expression Levels and Patterns of Stem Cell Marker, Aldehyde Dehydrogenase Isoform 1 (ALDH1), in Human Epithelial Cancers. PLoS ONE.

[38] Skoda J., Hermanova M., Loja T., Nemec P., Neradil J., Karasek P., Veselska R. (2016). Co-Expression of Cancer Stem Cell Markers Corresponds to a Pro-Tumorigenic Expression Profile in Pancreatic Adenocarcinoma. PLoS ONE.

[39] Gupta V.K., Sharma N.S., Kesh K., Dauer P., Nomura A., Giri B., Dudeja V., Banerjee S., Bhattacharya S., Saluja A. (2018). Metastasis and Chemoresistance in CD133 Expressing Pancreatic Cancer Cells Are Dependent on Their Lipid Raft Integrity. Cancer Lett..

[40] Ren C., Chen H., Han C., Wang D., Fu D. (2012). Increased Plasma MicroRNA and CD133/CK18-positive Cancer Cells in the Pleural Fluid of a Pancreatic Cancer Patient with Liver and Pleural Metastases and Correlation with Chemoresistance. Oncol. Lett..

[41] Mueller M., Hermann P.C., Witthauer J., Rubio–Viqueira B., Leicht S.F., Huber S., Ellwart J.W., Mustafa M., Bartenstein P., D’Haese J.G. (2009). Combined Targeted Treatment to Eliminate Tumorigenic Cancer Stem Cells in Human Pancreatic Cancer. Gastroenterology.

[42] Kise K., Kinugasa-Katayama Y., Takakura N. (2016). Tumor Microenvironment for Cancer Stem Cells. Adv. Drug. Deliv. Rev..

[43] Bocci F., Gearhart-Serna L., Boareto M., Ribeiro M., Ben-Jacob E., Devi G.R., Levine H., Onuchic J.N., Jolly M.K. (2019). Toward Understanding Cancer Stem Cell Heterogeneity in the Tumor Microenvironment. Proc. Natl. Acad. Sci. USA.

[44] Begum A., McMillan R.H., Chang Y.-T., Penchev V.R., Maitra A., Goggins M.G., Eshelman J.R., Wolfgang C.L., Rasheed Z.A. (2019). Direct Interactions With Cancer-Associated Fibroblasts Lead to Enhanced Pancreatic Cancer Stem Cell Function. Pancreas.

[45] Yang S., Wang X., Contino G., Liesa M., Sahin E., Ying H., Bause A., Li Y., Stommel J.M., Dell’Antonio G. (2011). Pancreatic Cancers Require Autophagy for Tumor Growth. Genes Dev..

[46] Song B., Bian Q., Zhang Y.-J., Shao C.-H., Li G., Liu A.-A., Jing W., Liu R., Zhou Y.-Q., Jin G. (2015). Downregulation of ASPP2 in Pancreatic Cancer Cells Contributes to Increased Resistance to Gemcitabine through Autophagy Activation. Mol. Cancer.

[47] Yuen C.A., Asuthkar S., Guda M.R., Tsung A.J., Velpula K.K. (2016). Cancer Stem Cell Molecular Reprogramming of the Warburg Effect in Glioblastomas: A New Target Gleaned from an Old Concept. CNS Oncol..

[48] Fabian A., Stegner S., Miarka L., Zimmermann J., Lenk L., Rahn S., Buttlar J., Viol F., Knaack H., Esser D. (2019). Metastasis of Pancreatic Cancer: An Uninflamed Liver Micromilieu Controls Cell Growth and Cancer Stem Cell Properties by Oxidative Phosphorylation in Pancreatic Ductal Epithelial Cells. Cancer Lett..

[49] Cros J., Raffenne J., Couvelard A., Poté N. (2018). Tumor Heterogeneity in Pancreatic Adenocarcinoma. PAT.

[50] di Magliano M.P., Logsdon C.D. (2013). Roles for KRAS in Pancreatic Tumor Development and Progression. Gastroenterology.

[51] Kulemann B., Liss A.S., Warshaw A.L., Seifert S., Bronsert P., Glatz T., Pitman M.B., Hoeppner J. (2016). KRAS Mutations in Pancreatic Circulating Tumor Cells: A Pilot Study. Tumor Biol..

[52] Guo S., Shi X., Shen J., Gao S., Wang H., Shen S., Pan Y., Li B., Xu X., Shao Z. (2020). Preoperative Detection of KRAS G12D Mutation in CtDNA Is a Powerful Predictor for Early Recurrence of Resectable PDAC Patients. Br. J. Cancer.

[53] Morton J.P., Timpson P., Karim S.A., Ridgway R.A., Athineos D., Doyle B., Jamieson N.B., Oien K.A., Lowy A.M., Brunton V.G. (2010). Mutant P53 Drives Metastasis and Overcomes Growth Arrest/Senescence in Pancreatic Cancer. Proc. Natl. Acad. Sci. USA.

[54] Haugk B. (2010). Pancreatic Intraepithelial Neoplasia—Can We Detect Early Pancreatic Cancer?. Histopathology.

[55] Bailey J.M., Hendley A.M., Lafaro K.J., Pruski M.A., Jones N.C., Alsina J., Younes M., Maitra A., McAllister F., Iacobuzio-Donahue C.A. (2016). P53 Mutations Cooperate with Oncogenic Kras to Promote Adenocarcinoma from Pancreatic Ductal Cells. Oncogene.

[56] Bünger S., Barow M., Thorns C., Freitag-Wolf S., Danner S., Tiede S., Pries R., Görg S., Bruch H.-P., Roblick U.J. (2012). Pancreatic Carcinoma Cell Lines Reflect Frequency and Variability of Cancer Stem Cell Markers in Clinical Tissue. ESR.

[57] Sheikh A., Hussain S.A., Ghori Q., Naeem N., Fazil A., Giri S., Sathian B., Mainali P., Al Tamimi D.M. (2015). The Spectrum of Genetic Mutations in Breast Cancer. Asian Pac. J. Cancer Prev..

[58] Pellagatti A., Roy S., Di Genua C., Burns A., McGraw K., Valletta S., Larrayoz M.J., Fernandez-Mercado M., Mason J., Killick S. (2016). Targeted Resequencing Analysis of 31 Genes Commonly Mutated in Myeloid Disorders in Serial Samples from Myelodysplastic Syndrome Patients Showing Disease Progression. Leukemia.

[59] Maddipati R., Stanger B.Z. (2015). Pancreatic Cancer Metastases Harbor Evidence of Polyclonality. Cancer Discov..

[60] Ball C.R., Oppel F., Ehrenberg K.R., Dubash T.D., Dieter S.M., Hoffmann C.M., Abel U., Herbst F., Koch M., Werner J. (2017). Succession of Transiently Active Tumor-Initiating Cell Clones in Human Pancreatic Cancer Xenografts. Embo Mol. Med..

[61] Seth S., Li C.-Y., Ho I.-L., Corti D., Loponte S., Sapio L., Del Poggetto E., Yen E.-Y., Robinson F.S., Peoples M. (2019). Pre-Existing Functional Heterogeneity of Tumorigenic Compartment as the Origin of Chemoresistance in Pancreatic Tumors. Cell Rep..

[62] Saikrishna L., Kasa P., Momin S., Bhaskar L.V.K.S., Nagaraju G.P., BM Reddy A. (2019). Perspectives and Molecular Understanding of Pancreatic Cancer Stem Cells. Exploring Pancreatic Metabolism and Malignancy.

[63] Ikenaga N., Ohuchida K., Mizumoto K., Yu J., Kayashima T., Hayashi A., Nakata K., Tanaka M. (2010). Characterization of CD24 Expression in Intraductal Papillary Mucinous Neoplasms and Ductal Carcinoma of the Pancreas. Hum. Pathol..

[64] Hermann P.C., Huber S.L., Herrler T., Aicher A., Ellwart J.W., Guba M., Bruns C.J., Heeschen C. (2007). Distinct Populations of Cancer Stem Cells Determine Tumor Growth and Metastatic Activity in Human Pancreatic Cancer. Cell Stem Cell.

[65] D’Alterio C., Cindolo L., Portella L., Polimeno M., Consales C., Riccio A., Cioffi M., Franco R., Chiodini P., Cartenì G. (2010). Differential Role of CD133 and CXCR4 in Renal Cell Carcinoma. Cell Cycle.

[66] Bertolini G., D’Amico L., Moro M., Landoni E., Perego P., Miceli R., Gatti L., Andriani F., Wong D., Caserini R. (2015). Microenvironment-Modulated Metastatic CD133+/CXCR4+/EpCAM− Lung Cancer–Initiating Cells Sustain Tumor Dissemination and Correlate with Poor Prognosis. Cancer Res..

[67] Sun Y., Yoshida T., Okabe M., Zhou K., Wang F., Soko C., Saito S., Nikaido T. (2017). Isolation of Stem-Like Cancer Cells in Primary Endometrial Cancer Using Cell Surface Markers CD133 and CXCR4. Transl. Oncol..

[68] Sancho P., Alcala S., Usachov V., Hermann P.C., Sainz B. (2016). The Ever-Changing Landscape of Pancreatic Cancer Stem Cells. Pancreatology.

[69] Sugiyama T., Shuto T., Suzuki S., Sato T., Koga T., Suico M.A., Kusuhara H., Sugiyama Y., Cyr D.M., Kai H. (2011). Posttranslational Negative Regulation of Glycosylated and Non-Glycosylated BCRP Expression by Derlin-1. Biochem. Biophys. Res. Commun..

[70] Murtaugh L.C., Stanger B.Z., Kwan K.M., Melton D.A. (2003). Notch Signaling Controls Multiple Steps of Pancreatic Differentiation. Proc. Natl. Acad. Sci. USA.

[71] Sharon N., Vanderhooft J., Straubhaar J., Mueller J., Chawla R., Zhou Q., Engquist E.N., Trapnell C., Gifford D.K., Melton D.A. (2019). Wnt Signaling Separates the Progenitor and Endocrine Compartments during Pancreas Development. Cell Rep..

[72] Hebrok M., Kim S.K., Jacques B.S., McMahon A.P., Melton D.A. (2000). Regulation of Pancreas Development by Hedgehog Signaling. Development.

[73] Reichrath J., Reichrath S. (2012). Notch Signaling in Embryology and Cancer. Advances in Experimental Medicine and Biology.

[74] Mullendore M.E., Koorstra J.-B., Li Y.-M., Offerhaus G.J., Fan X., Henderson C.M., Matsui W., Eberhart C.G., Maitra A., Feldmann G. (2009). Ligand-Dependent Notch Signaling Is Involved in Tumor Initiation and Tumor Maintenance in Pancreatic Cancer. Clin. Cancer Res..

[75] Wang Z., Ahmad A., Li Y., Azmi A.S., Miele L., Sarkar F.H. (2011). Targeting Notch to Eradicate Pancreatic Cancer Stem Cells for Cancer Therapy. Anticancer Res..

[76] Abel E.V., Kim E.J., Wu J., Hynes M., Bednar F., Proctor E., Wang L., Dziubinski M.L., Simeone D.M. (2014). The Notch Pathway Is Important in Maintaining the Cancer Stem Cell Population in Pancreatic Cancer. PLoS ONE.

[77] Yabuuchi S., Pai S.G., Campbell N.R., de Wilde R.F., De Oliveira E., Korangath P., Streppel M.M., Rasheed Z.A., Hidalgo M., Maitra A. (2013). Notch Signaling Pathway Targeted Therapy Suppresses Tumor Progression and Metastatic Spread in Pancreatic Cancer. Cancer Lett..

[78] Lee J.Y., Song S.Y., Park J.Y. (2014). Notch Pathway Activation Is Associated with Pancreatic Cancer Treatment Failure. Pancreatology.

[79] Mizuma M., Rasheed Z.A., Yabuuchi S., Omura N., Campbell N.R., de Wilde R.F., Oliveira E.D., Zhang Q., Puig O., Matsui W. (2012). The Gamma Secretase Inhibitor MRK-003 Attenuates Pancreatic Cancer Growth in Preclinical Models. Mol. Cancer.

[80] Aggarwal B.B., Kumar A., Bharti A.C. (2003). Anticancer Potential of Curcumin: Preclinical and Clinical Studies. Anticancer Res..

[81] Bimonte S., Barbieri A., Leongito M., Piccirillo M., Giudice A., Pivonello C., De Angelis C., Granata V., Palaia R., Izzo F. (2016). Curcumin AntiCancer Studies in Pancreatic Cancer. Nutrients.

[82] Abbas Momtazi A., Sahebkar A. (2016). Difluorinated Curcumin: A Promising Curcumin Analogue with Improved Anti-Tumor Activity and Pharmacokinetic Profile. Curr. Pharm. Des..

[83] Xia J., Duan Q., Ahmad A., Bao B., Banerjee S., Shi Y., Ma J., Geng J., Chen Z., Wahidur Rahman K. (2012). Genistein Inhibits Cell Growth and Induces Apoptosis Through Up-Regulation of MiR-34a in Pancreatic Cancer Cells. Curr. Drug Targets.

[84] Nwaeburu C.C., Abukiwan A., Zhao Z., Herr I. (2017). Quercetin-Induced MiR-200b-3p Regulates the Mode of Self-Renewing Divisions in Pancreatic Cancer. Mol. Cancer.

[85] Pham N.-A., Jacobberger J.W., Schimmer A.D., Cao P., Gronda M., Hedley D.W. (2004). The Dietary Isothiocyanate Sulforaphane Targets Pathways of Apoptosis, Cell Cycle Arrest, and Oxidative Stress in Human Pancreatic Cancer Cells and Inhibits Tumor Growth in Severe Combined Immunodeficient Mice. Mol. Cancer.

[86] Appari M., Babu K.R., Kaczorowski A., Gross W., Herr I. (2014). Sulforaphane, Quercetin and Catechins Complement Each Other in Elimination of Advanced Pancreatic Cancer by MiR-Let-7 Induction and K-Ras Inhibition. Int. J. Oncol..

[87] Srivastava R.K., Tang S.-N., Zhu W., Meeker D., Shankar S. (2011). Sulforaphane Synergizes with Quercetin to Inhibit Self-Renewal Capacity of Pancreatic Cancer Stem Cells. Front. Biosci (Elite Ed.).

[88] Kallifatidis G., Labsch S., Rausch V., Mattern J., Gladkich J., Moldenhauer G., Büchler M.W., Salnikov A.V., Herr I. (2011). Sulforaphane Increases Drug-Mediated Cytotoxicity Toward Cancer Stem-like Cells of Pancreas and Prostate. Mol. Ther..

[89] Seidensticker M.J., Behrens J. (2000). Biochemical Interactions in the Wnt Pathway. Biochim. Biophys. Acta (BBA) Mol. Cell Res..

[90] Polakis P. (2000). Wnt Signaling and Cancer. Genes Dev..

[91] di Magliano M.P., Biankin A.V., Heiser P.W., Cano D.A., Gutierrez P.J.A., Deramaudt T., Segara D., Dawson A.C., Kench J.G., Henshall S.M. (2007). Common Activation of Canonical Wnt Signaling in Pancreatic Adenocarcinoma. PLoS ONE.

[92] Curtin J.C., Lorenzi M.V. (2010). Drug Discovery Approaches to Target Wnt Signaling in Cancer Stem Cells. Oncotarget.

[93] He L., Wang F., Dai W.-Q., Wu D., Lin C.-L., Wu S.-M., Cheng P., Zhang Y., Shen M., Wang C.-F. (2013). Mechanism of Action of Salinomycin on Growth and Migration in Pancreatic Cancer Cell Lines. Pancreatology.

[94] Zhang G.-N., Liang Y., Zhou L.-J., Chen S.-P., Chen G., Zhang T.-P., Kang T., Zhao Y.-P. (2011). Combination of Salinomycin and Gemcitabine Eliminates Pancreatic Cancer Cells. Cancer Lett..

[95] Lamb R., Ozsvari B., Lisanti C.L., Tanowitz H.B., Howell A., Martinez-Outschoorn U.E., Sotgia F., Lisanti M.P. (2015). Antibiotics That Target Mitochondria Effectively Eradicate Cancer Stem Cells, across Multiple Tumor Types: Treating Cancer like an Infectious Disease. Oncotarget.

[96] Lu Z., Xu N., He B., Pan C., Lan Y., Zhou H., Liu X. (2017). Inhibition of Autophagy Enhances the Selective Anti-Cancer Activity of Tigecycline to Overcome Drug Resistance in the Treatment of Chronic Myeloid Leukemia. J. Exp. Clin. Cancer Res..

[97] Li H., Jiao S., Li X., Banu H., Hamal S., Wang X. (2015). Therapeutic Effects of Antibiotic Drug Tigecycline against Cervical Squamous Cell Carcinoma by Inhibiting Wnt/β-Catenin Signaling. Biochem. Biophys. Res. Commun..

[98] Dong Z., Abbas M.N., Kausar S., Yang J., Li L., Tan L., Cui H. (2019). Biological Functions and Molecular Mechanisms of Antibiotic Tigecycline in the Treatment of Cancers. Int. J. Mol. Sci..

[99] Yang J., Dong Z., Ren A., Fu G., Zhang K., Li C., Wang X., Cui H. (2020). Antibiotic Tigecycline Inhibits Cell Proliferation, Migration and Invasion via down-Regulating CCNE2 in Pancreatic Ductal Adenocarcinoma. J. Cell. Mol. Med..

[100] Sahib A.K., Loureiro J.R., Vasavada M., Anderson C., Kubicki A., Wade B., Joshi S.H., Woods R.P., Congdon E., Espinoza R. (2020). Modulation of the Functional Connectome in Major Depressive Disorder by Ketamine Therapy. Psychol. Med..

[101] Hu J., Duan W., Liu Y. (2020). Ketamine Inhibits Aerobic Glycolysis in Colorectal Cancer Cells by Blocking the NMDA Receptor-CaMK II-c-Myc Pathway. Clin. Exp. Pharmacol. Physiol..

[102] Carbone C., Piro G., Gaianigo N., Ligorio F., Santoro R., Merz V., Simionato F., Zecchetto C., Falco G., Conti G. (2018). Adipocytes Sustain Pancreatic Cancer Progression through a Non-Canonical WNT Paracrine Network Inducing ROR2 Nuclear Shuttling. Int. J. Obes..

[103] Davis S.L., Cardin D.B., Shahda S., Lenz H.-J., Dotan E., O’Neil B.H., Kapoun A.M., Stagg R.J., Berlin J., Messersmith W.A. (2020). A Phase 1b Dose Escalation Study of Wnt Pathway Inhibitor Vantictumab in Combination with Nab-Paclitaxel and Gemcitabine in Patients with Previously Untreated Metastatic Pancreatic Cancer. Investig. New Drugs.

[104] Cohen M.M. (2003). The Hedgehog Signaling Network. Am. J. Med. Genet. Part A.

[105] Varjosalo M., Taipale J. (2008). Hedgehog: Functions and Mechanisms. Genes Dev..

[106] Thayer S.P., di Magliano M.P., Heiser P.W., Nielsen C.M., Roberts D.J., Lauwers G.Y., Qi Y.P., Gysin S., Castillo C.F., Yajnik V. (2003). Hedgehog Is an Early and Late Mediator of Pancreatic Cancer Tumorigenesis. Nature.

[107] Mathew E., Collins M.A., Fernandez-Barrena M.G., Holtz A.M., Yan W., Hogan J.O., Tata Z., Allen B.L., Fernandez-Zapico M.E., Pasca di Magliano M. (2014). The Transcription Factor GLI1 Modulates the Inflammatory Response during Pancreatic Tissue Remodeling. J. Biol. Chem..

[108] Tang S.-N., Fu J., Nall D., Rodova M., Shankar S., Srivastava R.K. (2012). Inhibition of Sonic Hedgehog Pathway and Pluripotency Maintaining Factors Regulate Human Pancreatic Cancer Stem Cell Characteristics. Int. J. Cancer.

[109] Li X., Ma Q., Xu Q., Liu H., Lei J., Duan W., Bhat K., Wang F., Wu E., Wang Z. (2012). SDF-1/CXCR4 Signaling Induces Pancreatic Cancer Cell Invasion and Epithelial–Mesenchymal Transition in Vitro through Non-Canonical Activation of Hedgehog Pathway. Cancer Lett..

[110] Merchant A.A., Matsui W. (2010). Targeting Hedgehog—A Cancer Stem Cell Pathway. Clin. Cancer Res..

[111] Chen J.K., Taipale J., Cooper M.K., Beachy P.A. (2002). Inhibition of Hedgehog Signaling by Direct Binding of Cyclopamine to Smoothened. Genes Dev..

[112] Xu X.-F., Guo C.-Y., Liu J., Yang W.-J., Xia Y.-J., Xu L., Yu Y.-C., Wang X.-P. (2009). Gli1 Maintains Cell Survival by Up-Regulating IGFBP6 and Bcl-2 through Promoter Regions in Parallel Manner in Pancreatic Cancer Cells. J. Carcinog..

[113] Huynh D.L., Koh H., Chandimali N., Zhang J.J., Kim N., Kang T.Y., Ghosh M., Gera M., Park Y.-H., Kwon T. (2019). BRM270 Inhibits the Proliferation of CD44 Positive Pancreatic Ductal Adenocarcinoma Cells via Downregulation of Sonic Hedgehog Signaling. Evid. Based Complement. Altern. Med..

[114] Huang F.-T., Zhuan-Sun Y.-X., Zhuang Y.-Y., Wei S.-L., Tang J., Chen W.-B., Zhang S.-N. (2012). Inhibition of Hedgehog Signaling Depresses Self-Renewal of Pancreatic Cancer Stem Cells and Reverses Chemoresistance. Int. J. Oncol..

[115] Balic A., Sørensen M.D., Trabulo S.M., Sainz B., Cioffi M., Vieira C.R., Miranda-Lorenzo I., Hidalgo M., Kleeff J., Erkan M. (2014). Chloroquine Targets Pancreatic Cancer Stem Cells via Inhibition of CXCR4 and Hedgehog Signaling. Mol. Cancer.

[116] Rangarajan P., Subramaniam D., Paul S., Kwatra D., Palaniyandi K., Islam S., Harihar S., Ramalingam S., Gutheil W., Putty S. (2015). Crocetinic Acid Inhibits Hedgehog Signaling to Inhibit Pancreatic Cancer Stem Cells. Oncotarget.

[117] Sun X.-D., Liu X.-E., Huang D.-S. (2013). Curcumin Reverses the Epithelial-Mesenchymal Transition of Pancreatic Cancer Cells by Inhibiting the Hedgehog Signaling Pathway. Oncol. Rep..

[118] Cao L., Xiao X., Lei J., Duan W., Ma Q., Li W. (2016). Curcumin Inhibits Hypoxia-Induced Epithelial-mesenchymal Transition in Pancreatic Cancer Cells via Suppression of the Hedgehog Signaling Pathway. Oncol. Rep..

[119] Li S.-H., Fu J., Watkins D.N., Srivastava R.K., Shankar S. (2013). Sulforaphane Regulates Self-Renewal of Pancreatic Cancer Stem Cells through the Modulation of Sonic Hedgehog–GLI Pathway. Mol. Cell Biochem..

[120] Feldmann G., Fendrich V., McGovern K., Bedja D., Bisht S., Alvarez H., Koorstra J.-B.M., Habbe N., Karikari C., Mullendore M. (2008). An Orally Bioavailable Small-Molecule Inhibitor of Hedgehog Signaling Inhibits Tumor Initiation and Metastasis in Pancreatic Cancer. Mol. Cancer.

[121] Singh B.N., Fu J., Srivastava R.K., Shankar S. (2011). Hedgehog Signaling Antagonist GDC-0449 (Vismodegib) Inhibits Pancreatic Cancer Stem Cell Characteristics: Molecular Mechanisms. PLoS ONE.

[122] Kim E.J., Sahai V., Abel E.V., Griffith K.A., Greenson J.K., Takebe N., Khan G.N., Blau J.L., Craig R., Balis U.G. (2014). Pilot Clinical Trial of Hedgehog Pathway Inhibitor GDC-0449 (Vismodegib) in Combination with Gemcitabine in Patients with Metastatic Pancreatic Adenocarcinoma. Clin. Cancer Res..

[123] Misra J.R., Irvine K.D. (2018). The Hippo Signaling Network and Its Biological Functions. Annu. Rev. Genet..

[124] Pan D. (2010). The Hippo Signaling Pathway in Development and Cancer. Dev. Cell.

[125] Ansari D., Ohlsson H., Althini C., Bauden M., Zhou Q., Hu D., Andersson R. (2019). The Hippo Signaling Pathway in Pancreatic Cancer. Anticancer Res..

[126] Zhang L., Shi H., Chen H., Gong A., Liu Y., Song L., Xu X., You T., Fan X., Wang D. (2019). Dedifferentiation Process Driven by Radiotherapy-Induced HMGB1/TLR2/YAP/HIF-1α Signaling Enhances Pancreatic Cancer Stemness. Cell Death Dis..

[127] Santoro R., Zanotto M., Carbone C., Piro G., Tortora G., Melisi D. (2018). MEKK3 Sustains EMT and Stemness in Pancreatic Cancer by Regulating YAP and TAZ Transcriptional Activity. Anticancer Res..

[128] Chen W., Wang H., Liu Y., Xu W., Ling C., Li Y., Liu J., Chen M., Zhang Y., Chen B. (2020). Linc-RoR Promotes Proliferation, Migration, and Invasion via the Hippo/YAP Pathway in Pancreatic Cancer Cells. J. Cell. Biochem..

[129] Gruber R., Panayiotou R., Nye E., Spencer-Dene B., Stamp G., Behrens A. (2016). YAP1 and TAZ Control Pancreatic Cancer Initiation in Mice by Direct Up-Regulation of JAK–STAT3 Signaling. Gastroenterology.

[130] Zhang W., Nandakumar N., Shi Y., Manzano M., Smith A., Graham G., Gupta S., Vietsch E.E., Laughlin S.Z., Wadhwa M. (2014). Downstream of Mutant KRAS, the Transcription Regulator YAP Is Essential for Neoplastic Progression to Pancreatic Ductal Adenocarcinoma. Sci. Signal..

[131] Zhao X., Wang X., Fang L., Lan C., Zheng X., Wang Y., Zhang Y., Han X., Liu S., Cheng K. (2017). A Combinatorial Strategy Using YAP and Pan-RAF Inhibitors for Treating KRAS-Mutant Pancreatic Cancer. Cancer Lett..

[132] Yu H., Lee H., Herrmann A., Buettner R., Jove R. (2014). Revisiting STAT3 Signalling in Cancer: New and Unexpected Biological Functions. Nat. Rev. Cancer.

[133] Johnson D.E., O’Keefe R.A., Grandis J.R. (2018). Targeting the IL-6/JAK/STAT3 Signalling Axis in Cancer. Nat. Rev. Clin. Oncol..

[134] Corcoran R.B., Contino G., Deshpande V., Tzatsos A., Conrad C., Benes C.H., Levy D.E., Settleman J., Engelman J.A., Bardeesy N. (2011). STAT3 Plays a Critical Role in KRAS-Induced Pancreatic Tumorigenesis. Cancer Res..

[135] Zhang Z., Duan Q., Zhao H., Liu T., Wu H., Shen Q., Wang C., Yin T. (2016). Gemcitabine Treatment Promotes Pancreatic Cancer Stemness through the Nox/ROS/NF-ΚB/STAT3 Signaling Cascade. Cancer Lett..

[136] Liu X., Wang J., Wang H., Yin G., Liu Y., Lei X., Xiang M. (2015). REG3A Accelerates Pancreatic Cancer Cell Growth under IL-6-Associated Inflammatory Condition: Involvement of a REG3A–JAK2/STAT3 Positive Feedback Loop. Cancer Lett..

[137] Palagani V., Bozko P., El Khatib M., Belahmer H., Giese N., Sipos B., Malek N.P., Plentz R.R. (2014). Combined Inhibition of Notch and JAK/STAT Is Superior to Monotherapies and Impairs Pancreatic Cancer Progression. Carcinogenesis.

[138] Lian J.P., Word B., Taylor S., Hammons G.J., Lyn-Cook B.D. (2004). Modulation of the Constitutive Activated STAT3 Transcription Factor in Pancreatic Cancer Prevention: Effects of Indole-3-Carbinol (I3C) and Genistein. Anticancer Res..

[139] Glienke W., Maute L., Wicht J., Bergmann L. (2009). Curcumin Inhibits Constitutive STAT3 Phosphorylation in Human Pancreatic Cancer Cell Lines and Downregulation of Survivin/BIRC5 Gene Expression. Cancer Investig..

[140] Duan J., Yue W., JianYu E., Malhotra J., Lu S., Gu J., Xu F., Tan X.-L. (2016). In Vitro Comparative Studies of Resveratrol and Triacetylresveratrol on Cell Proliferation, Apoptosis, and STAT3 and NFκB Signaling in Pancreatic Cancer Cells. Sci. Rep..

[141] Sharma N., Nanta R., Sharma J., Gunewardena S., Singh K.P., Shankar S., Srivastava R.K. (2015). PI3K/AKT/MTOR and Sonic Hedgehog Pathways Cooperate Together to Inhibit Human Pancreatic Cancer Stem Cell Characteristics and Tumor Growth. Oncotarget.

[142] Husain K., Centeno B.A., Coppola D., Trevino J., Sebti S.M., Malafa M.P. (2017). δ-Tocotrienol, a Natural Form of Vitamin E, Inhibits Pancreatic Cancer Stem-like Cells and Prevents Pancreatic Cancer Metastasis. Oncotarget.

[143] Shin-Kang S., Ramsauer V.P., Lightner J., Chakraborty K., Stone W., Campbell S., Reddy S.A.G., Krishnan K. (2011). Tocotrienols Inhibit AKT and ERK Activation and Suppress Pancreatic Cancer Cell Proliferation by Suppressing the ErbB2 Pathway. Free Radic. Biol. Med..

[144] Yang K., Li Y., Lian G., Lin H., Shang C., Zeng L., Chen S., Li J., Huang C., Huang K. (2018). KRAS Promotes Tumor Metastasis and Chemoresistance by Repressing RKIP via the MAPK–ERK Pathway in Pancreatic Cancer. Int. J. Cancer.

[145] Ding W., Mouzaki M., You H., Laird J.C., Mato J., Lu S.C., Rountree C.B. (2009). CD133+ Liver Cancer Stem Cells from Methionine Adenosyl Transferase 1A–Deficient Mice Demonstrate Resistance to Transforming Growth Factor (TGF)-β–Induced Apoptosis. Hepatology.

[146] Zhang L., Wang D., Li Y., Liu Y., Xie X., Wu Y., Zhou Y., Ren J., Zhang J., Zhu H. (2016). CCL21/CCR7 Axis Contributed to CD133+ Pancreatic Cancer Stem-Like Cell Metastasis via EMT and Erk/NF-ΚB Pathway. PLoS ONE.

[147] Chai X., Chu H., Yang X., Meng Y., Shi P., Gou S. (2015). Metformin Increases Sensitivity of Pancreatic Cancer Cells to Gemcitabine by Reducing CD133 + Cell Populations and Suppressing ERK/P70S6K Signaling. Sci. Rep..

[148] Mori T., Doi R., Koizumi M., Toyoda E., Ito D., Kami K., Masui T., Fujimoto K., Tamamura H., Hiramatsu K. (2004). CXCR4 Antagonist Inhibits Stromal Cell-Derived Factor 1-Induced Migration and Invasion of Human Pancreatic Cancer. Mol. Cancer.

[149] Gelmini S., Mangoni M., Serio M., Romagnani P., Lazzeri E. (2008). The Critical Role of SDF-1/CXCR4 Axis in Cancer and Cancer Stem Cells Metastasis. J. Endocrinol. Investig..

[150] Fitzgerald T.L., McCubrey J.A. (2014). Pancreatic Cancer Stem Cells: Association with Cell Surface Markers, Prognosis, Resistance, Metastasis and Treatment. Adv. Biol. Regul..

[151] Wang H., Tsang B.K. (2007). Nodal Signalling and Apoptosis. Reproduction.

[152] Lonardo E., Hermann P.C., Mueller M.-T., Huber S., Balic A., Miranda-Lorenzo I., Zagorac S., Alcala S., Rodriguez-Arabaolaza I., Ramirez J.C. (2011). Nodal/Activin Signaling Drives Self-Renewal and Tumorigenicity of Pancreatic Cancer Stem Cells and Provides a Target for Combined Drug Therapy. Cell Stem Cell.

[153] Wei H., Wang F., Wang Y., Li T., Xiu P., Zhong J., Sun X., Li J. (2017). Verteporfin Suppresses Cell Survival, Angiogenesis and Vasculogenic Mimicry of Pancreatic Ductal Adenocarcinoma via Disrupting the YAP-TEAD Complex. Cancer Sci..

[154] Bloomston M., Frankel W.L., Petrocca F., Volinia S., Alder H., Hagan J.P., Liu C.-G., Bhatt D., Taccioli C., Croce C.M. (2007). MicroRNA Expression Patterns to Differentiate Pancreatic Adenocarcinoma From Normal Pancreas and Chronic Pancreatitis. JAMA.

[155] Lee E.J., Gusev Y., Jiang J., Nuovo G.J., Lerner M.R., Frankel W.L., Morgan D.L., Postier R.G., Brackett D.J., Schmittgen T.D. (2007). Expression Profiling Identifies MicroRNA Signature in Pancreatic Cancer. Int. J. Cancer.

[156] Schultz N.A., Dehlendorff C., Jensen B.V., Bjerregaard J.K., Nielsen K.R., Bojesen S.E., Calatayud D., Nielsen S.E., Yilmaz M., Holländer N.H. (2014). MicroRNA Biomarkers in Whole Blood for Detection of Pancreatic Cancer. JAMA.

[157] Reddy K.B. (2015). MicroRNA (MiRNA) in Cancer. Cancer Cell Int..

[158] Singh S., Chitkara D., Kumar V., Behrman S.W., Mahato R.I. (2013). MiRNA Profiling in Pancreatic Cancer and Restoration of Chemosensitivity. Cancer Lett..

[159] Bao B., Ali S., Banerjee S., Wang Z., Logna F., Azmi A.S., Kong D., Ahmad A., Li Y., Padhye S. (2012). Curcumin Analogue CDF Inhibits Pancreatic Tumor Growth by Switching on Suppressor MicroRNAs and Attenuating EZH2 Expression. Cancer Res..

[160] Hong T.H., Park I.Y. (2014). MicroRNA Expression Profiling of Diagnostic Needle Aspirates from Surgical Pancreatic Cancer Specimens. Ann. Surg Treat. Res..

[161] Schultz N.A., Werner J., Willenbrock H., Roslind A., Giese N., Horn T., Wøjdemann M., Johansen J.S. (2012). MicroRNA Expression Profiles Associated with Pancreatic Adenocarcinoma and Ampullary Adenocarcinoma. Mod. Pathol..

[162] Calatayud D., Dehlendorff C., Boisen M.K., Hasselby J.P., Schultz N.A., Werner J., Immervoll H., Molven A., Hansen C.P., Johansen J.S. (2017). Tissue MicroRNA Profiles as Diagnostic and Prognostic Biomarkers in Patients with Resectable Pancreatic Ductal Adenocarcinoma and Periampullary Cancers. Biomark Res..

[163] Papaconstantinou I.G., Manta A., Gazouli M., Lyberopoulou A., Lykoudis P.M., Polymeneas G., Voros D. (2013). Expression of MicroRNAs in Patients with Pancreatic Cancer and Its Prognostic Significance. Pancreas.

[164] Morimura R., Komatsu S., Ichikawa D., Takeshita H., Tsujiura M., Nagata H., Konishi H., Shiozaki A., Ikoma H., Okamoto K. (2011). Novel Diagnostic Value of Circulating MiR-18a in Plasma of Patients with Pancreatic Cancer. Br. J. Cancer.

[165] Olson P., Lu J., Zhang H., Shai A., Chun M.G., Wang Y., Libutti S.K., Nakakura E.K., Golub T.R., Hanahan D. (2009). MicroRNA Dynamics in the Stages of Tumorigenesis Correlate with Hallmark Capabilities of Cancer. Genes Dev..

[166] Hu Y., Ou Y., Wu K., Chen Y., Sun W. (2012). MiR-143 Inhibits the Metastasis of Pancreatic Cancer and an Associated Signaling Pathway. Tumour Biol..

[167] Frampton A.E., Krell J., Jacob J., Stebbing J., Castellano L., Jiao L.R. (2012). Loss of MiR-126 Is Crucial to Pancreatic Cancer Progression. Expert Rev. Anticancer.

[168] Li Y., Vandenboom T.G., Wang Z., Kong D., Ali S., Philip P.A., Sarkar F.H. (2010). MiR-146a Suppresses Invasion of Pancreatic Cancer Cells. Cancer Res..

[169] He H., Di Y., Liang M., Yang F., Yao L., Hao S., Li J., Jiang Y., Jin C., Fu D. (2013). The MicroRNA-218 and ROBO-1 Signaling Axis Correlates with the Lymphatic Metastasis of Pancreatic Cancer. Oncol. Rep..

[170] He H., Hao S.-J., Yao L., Yang F., Di Y., Li J., Jiang Y.-J., Jin C., Fu D.-L. (2014). MicroRNA-218 Inhibits Cell Invasion and Migration of Pancreatic Cancer via Regulating ROBO1. Cancer Biol..

[171] Yuan Q., Zhang Y., Li J., Cao G., Yang W. (2018). High Expression of MicroRNA-4295 Contributes to Cell Proliferation and Invasion of Pancreatic Ductal Adenocarcinoma by the down-Regulation of Glypican-5. Biochem. Biophys. Res. Commun..

